# Vertical Barriers for Land Contamination Containment: A Review

**DOI:** 10.3390/ijerph182312643

**Published:** 2021-11-30

**Authors:** Benyi Cao, Jian Xu, Fei Wang, Yunhui Zhang, David O’Connor

**Affiliations:** 1 State Environmental Protection Key Laboratory of Soil Environmental Management and Pollution Control, Nanjing Institute of Environmental Sciences, Ministry of Ecology and Environment of China, Nanjing 210042, China; caobenyi@outlook.com; 2 Department of Engineering, University of Cambridge, Cambridge CB2 1PZ, UK; 3 Institute of Geotechnical Engineering, School of Transportation, Southeast University, Nanjing 211102, China; 101012020@seu.edu.cn; 4 College of Environmental Science and Engineering, Tongji University, Shanghai 200092, China; yunhuizhang@tongji.edu.cn; 5 School of Real Estate & Land Management, Royal Agricultural University, Cirencester GL7 6JS, UK; David.OConnor@rau.ac.uk

**Keywords:** in-ground barrier, cut-off wall, resilient material, land contamination, soil pollution

## Abstract

Soil pollution is one of the major threats to the environment and jeopardizes the provision of key soil ecosystem services. Vertical barriers, including slurry trench walls and walls constructed with soil mix technology, have been employed for decades to control groundwater flow and subsurface contaminant transport. This paper comprehensively reviewed and assessed the typical materials and mechanical and permeability properties of soil–bentonite, cement–bentonite and soil mix barriers, with the values of mix design and engineering properties summarized and compared. In addition, the damage and durability of barrier materials under mechanical, chemical, and environmental stresses were discussed. A number of landmark remediation projects were documented to demonstrate the effectiveness of the use of barrier systems. Recent research about crack-resistant and self-healing barrier materials incorporating polymers and minerals at Cambridge University and performance monitoring techniques were analyzed. Future work should focus on two main areas: the use of geophysical methods for non-destructive monitoring and the optimization of resilient barrier materials.

## 1. Introduction

Industrial activities have increasingly been causing severe environmental impacts on air, water, and soil. Toxic chemicals, including heavy metals, herbicides, pesticides, and other organic contaminants, can be absorbed by human beings and lead to health problems when they are released into the environment [1,2,3]. The Food and Agriculture Organization of the United Nations (FAO) and United Nations Environment Programme (UNEP) have recently reported that soil pollution is one of the major threats to the environment and jeopardizes the provision of key soil ecosystem services, including the provision of safe and nutritious food, the availability of clean water, and the existence and conservation of soil biodiversity [4].

Since the industrial revolution, land contamination has posed a serious challenge to developed countries such as the UK, which has over 200,000 potential contaminated sites [5]. Land contamination can be even more severe in developing countries. In China, for instance, a government report showed that 16.1% of all tested soil samples were polluted, covering 6.3 million square kilometers [6]. It is, therefore, important to effectively manage contaminated sites and mitigate the threat posed to public health and the environment.

Land contamination risk management has grown from a niche field into a booming business, becoming a multi-billion dollar industry in many developed countries and a flourishing market in developing countries [7,8,9]. There are three primary risk management approaches by which the ‘source–pathway–receptor’ pollutant linkages can be broken: (1) source removal or soil cleaning via treatment; (2) pathway management; (3) modification of exposure of the receptor [10,11,12]. Generally, contaminated sites are treated by combining these three approaches. For example, source removal can be achieved by flushing out the contaminants or by treating the soil chemically, thermally, or biologically; pathway management involves containment technologies that can encapsulate the contaminants and prevent their further spread; modifying the exposure of the receptor can be achieved by choosing a use for the land in the future where exposure will be reduced.

Vertical barriers have been employed for decades to control groundwater flow and subsurface contaminant transport [13,14]. There are two types of in-ground barriers, namely active and passive. Active barriers are alternately called permeable reactive barriers (PRBs). They are subsurface structures made up of reactive and hydraulically permeable materials. As the contaminated groundwater flows through the barrier, it immobilizes the contaminants through degradation, sorption, or precipitation [15,16]. In contrast, passive in-ground barriers are impermeable (cut-off) walls, which are mostly constructed using geotechnical engineering techniques and employed to redirect the groundwater flow and to isolate the contaminated site [17,18,19]. There are many types of impermeable barriers, and they can generally be categorized based on the construction methods and materials. In the USA, soil–bentonite barriers are widely used for the containment of polluted sites [20]. In the UK, the most common type of vertical barriers are cement–bentonite slurry trench walls, and the past three decades have witnessed the increasing application of soil mix technology to barrier construction [21]. In China, barriers constructed with soil mix technology have also been adopted in many land contamination risk management projects.

This paper comprehensively reviewed and assessed the typical materials, and mechanical and permeability properties of soil–bentonite, cement–bentonite, and soil mix barriers. In addition, the damage and durability of barrier materials under mechanical, chemical, and environmental stresses were discussed. A number of landmark remediation projects were documented to demonstrate the effectiveness of the use of barrier systems. Recent research about crack-resistant and self-healing barrier materials incorporating polymers and minerals and performance monitoring techniques were analyzed. Future work should focus on two main areas: the use of geophysical methods for non-destructive monitoring and the optimization of resilient vertical barrier materials.

## 2. Mix Design and Engineering Properties

### 2.1. Soil–Bentonite Slurry Trench Barriers

Soil–bentonite slurry trench barriers have been extensively used in the USA since the 1970s. The first field trial of a 15 m-deep soil–bentonite slurry trench barrier as a diaphragm wall was conducted by the U.S. Army Corps of Engineers at Terminal Island in California [22]. The first slurry trench barrier for the containment of possible contamination was constructed by the Bachy Company in 1966 in France. It is very interesting to note that this slurry trench barrier was installed as a precaution in case there were spills from a refinery rather than in response to existing contamination, and in today’s terminology this would be described as a preventative measure for land contamination risk management in an active industrial site.

The typical bentonite slurry comprises 4 to 7% dry sodium bentonite and the remaining 93 to 97% water, the density reported is between 1.03 and 1.12 g/cm^3^ [23]. It should be noted that the solid content in the fresh bentonite slurry depends on the bentonite quality. For example, for bentonite with a higher liquid limit (or swell index), the target bentonite content could be relatively low. The fresh properties of the bentonite slurry can be examined using the indicator parameters of Marshall viscosity, density, and filtrate loss. Both sodium and calcium bentonite are hydrophilic and absorbent, although, generally, sodium bentonite expands more than calcium bentonite after the absorption of water. The hydraulic conductivity of a soil–bentonite slurry barrier is dependent on both soil gradation and the quantity of bentonite used in blending. Typical permeabilities of soil–bentonite barriers range from over 10^−7^ m/s in backfill composed primarily of coarse soils, to less than 10^−10^ m/s in backfill containing over 60% clay. In practice, hydraulic conductivity higher than 10^−9^ m/s is not recommended for soil–bentonite barrier applications. Recently, backfills consisting of polymer- or biopolymer-amended bentonite and soil have received global attention, as conventional bentonite has poor chemical compatibility, yielding hydraulic conductivity higher than 10^-9^ m/s when exposed to cation-laden or heavy metal-laden groundwater [24]. Because of its low hydraulic conductivity, the barrier can be used to severely restrict downgradient groundwater movement. This causes the water level on the upgradient side of the barrier to rise significantly compared to the downgradient side. Therefore, the soil–bentonite barrier should be designed to withstand the great hydraulic gradients.

The strength of soil–bentonite barriers is not usually of primary concern for contamination containment applications. These barriers are normally designed to be comparable in strength to the surrounding ground [25]. Evans and Ryan (2005) reported the undrained shear strength of the order of 5 to 20 kPa by conducting laboratory and in situ tests [26]. They confirmed that the shear strength continues to increase with time because of secondary consolidation or creep and the thixotropic nature of the bentonite.

A primary requirement for backfill material is that it contains suitable particle size distribution, preferably with between 20 and 40% of fine particles able to pass through a number 200 sieve. Some researchers advocate the use of well-graded soil requiring lesser bentonite content to achieve the targeted low hydraulic conductivity [17]. The well-graded soil contains fewer pores because the voids are filled with progressively finer material, resulting in low hydraulic conductivity. Thereby, it requires lesser bentonite content to reduce the hydraulic conductivity. Moreover, the components of a well-graded soil are relatively stable against chemical change. Hence, the well-graded backfill with less bentonite is more preferred than poorly graded backfill with high bentonite content. Such a need for good backfill material could, however, limit the application of soil–bentonite barriers.

### 2.2. Cement–Bentonite Slurry Trench Barriers

During construction, a trench is first excavated under a head of cement–bentonite slurry. The cement–bentonite slurry is prepared in situ by mixing cement with a pre-hydrated bentonite slurry just before its discharge into the trench [27]. The composition and mix design of the cement–bentonite slurry was frequently varied until the role of each ingredient was understood [22,28]. Unlike in the soil–bentonite slurry, bentonite minerals in cement–bentonite slurries dissolve and are basically undetectable in the hardened barrier. Moreover, carbonation and pozzolanic chemical reactions take place during the period of cement hydration and curing, resulting in the formation of secondary hydration products. It is argued by Evans et al. (2021) that the compatibility criteria traditionally applied to soil–bentonite barriers might fail to work for cement–bentonite barriers [28]. It is proposed that because of the onset of carbonation, the cement–bentonite material could deteriorate in the event of an acidic pollutant attack; in addition, the formation of pozzolanic hydration products could increase the sorption capacity of the barrier to contaminants, particularly heavy metals. In addition to the role of bentonite, the proper cement content also plays a critical part in the quality of the mixed slurry. An extremely low cement content is not able to deliver the required self-hardening nature, and a too-high cement content cannot achieve the required flowability and workability [22]. The variation of one ingredient can affect the properties such as hydraulic conductivity, strength, deformability, and chemical compatibility.

In traditional practice for cement–bentonite barriers, Portland cement (PC) has been the primary cementitious material. Recently, ground-granulated blast-furnace slag (GGBS), manufactured from a by-product of the iron-making industry, has become a more environmentally friendly cement substitute. Using one ton of GGBS reduces the embodied CO_2_ by approximately 900 kg, compared to using one ton of PC [29]. Opdyke and Evans (2005) investigated the effects of the addition of GGBS as a cement replacement on the hydraulic conductivity and mechanical properties of cement–bentonite mixes [30]. Without GGBS replacement, or even with GGBS content of up to 70%, the hydraulic conductivity values are in the range from 1 × 10^−7^ to 1 × 10^−8^ m/s, which is the typical value of cement–bentonite walls traditionally formulated without slag [31]. Although the construction method of this type of barrier is similar to that of soil–bentonite, the hydraulic conductivity of cement–bentonite backfills (at 28 days curing time) is usually higher than that of soil–bentonite barriers. The strength improves with increasing GGBS replacement, with a maximum strength achieved at 80% GGBS replacement. Therefore, it was proposed that the optimum GGBS replacement range would be from 70–90% to achieve the best performance of cement–bentonite walls in terms of strength, strain at failure, and hydraulic conductivity. More recently, novel supplementary cementitious materials, particularly alkali-activated slag, are often used to replace ordinary PC in large quantities to enhance mechanical properties and durability [32,33]. For example, a recent study has shown that MgO activated slag and bentonite slurry has a higher strength and lower hydraulic conductivity because an abundant formation of expansive hydration products (hydrotalcite phases) could occupy the voids in the cementitious matrix, resulting in a denser microstructure [14]. The proportions of the different ingredients were changed by different researchers and their ratios are shown in Table 1. The reported cement content by weight varied from 1 to 30%, bentonite content varied from 2.8 to 7.0%, and water content varied from 65 to 85.5%.

The water-to-binder ratio also plays an important role in the properties of cement–bentonite barriers. It has been reported that a low water-to-cement ratio (2.8) led to hydraulic conductivity of less than 1 × 10^−8^ m/s, while a high water-to-cement ratio (7.5) produced hydraulic conductivity of more than 1 × 10^−7^ m/s [23]. This may be because the low water-to-cement ratio has a high solid content, and, thus, lower porosity. Additionally, the bentonite content has a significant influence on the hydraulic conductivity. Approximately 4 to 5% of bentonite can reduce the hydraulic conductivity with a slight increase in strength [31]. The bentonite hydrates and adds viscosity that prevents excessive settlement of the cement particles until a sufficient set has occurred to lock them up. The increased content of other finer particles, including GGBS or fly ash, also inhibits the tendency for slurry bleeding [38].

The hardened properties of cement–bentonite slurry walls are a function of time, i.e., the cement hydration reaction. The hydraulic conductivity is reduced and the strength is increased as the cement hydrates over time [18]. Strength is often only used as a quality control check—typically the unconfined compression strength (UCS) due to the simplicity of the test [31]. However, in some cases, the strength should be measured using multiple methods with various results applied to different failure cases, e.g., undrained results applied to evaluation during an earthquake and drained results for long-term ground movement potential. Based on a large amount of drained test data, Joshi (2009) proposed three modes of failure in drained loading conditions considering age and confining pressure [39]. The first mode is ‘strain hardening’, where a specimen experiences continuous compression and strength increases due to large void reduction. The second mode of failure is described as ‘ductile’, where the deviator stress rises rapidly and then there is a non-linear and gentle increase in stress until a very large strain. The third mode of failure can be called ‘brittle’, where specimens exhibit linear and high stiffness until the peak strength and then completely collapse beyond the peak. However, tests for these properties are carried out in a laboratory on samples cast from the poured slurry and allowed to set. There are reasons to suspect that laboratory-derived values do not entirely reflect the properties in situ. To address these issues a series of field measurements of cement–bentonite barriers were carried out [40]. In general, the barrier was found to be much more permeable than the laboratory tests had indicated, a difference that appears to be due to the scale effect.

### 2.3. Soil Mix Technology Constructed Barriers

Vertical barriers constructed via deep soil mixing employ a soil treatment methodology by which in situ soil is blended and mixed with cementitious and other agents to create a barrier. Its potential in geo-environmental applications became evident in the 1980s in the USA when the Bureau of Reclamation first used soil mixing to construct an upstream cut-off wall at Jackson Lake Dam in Wyoming [41]. In 1995, soil mixing was first introduced to the UK for the remediation of contaminated land [42]. Based on a cement content in soil between 100 and 300 kg/m^3^ (dry weight), barriers constructed using this method typically have predesign values of UCS of more than 1 MPa and hydraulic conductivity of 1 × 10^−8^ m/s [41,43].

The grout mixtures normally consist of cement and bentonite as with a cement–bentonite slurry trench wall, although, with much lower water content. GGBS and fly ash are two main replacements that have been added to cement grout to reduce cement usage and improve durability. The addition of GGBS improves the hydrated cement product because it reduces the weak portlandite content and increases the quantity of stronger calcium silicate hydrate (CSH) [44]. Pulverized fuel ash (PFA) is a synthetic pozzolan created by the combustion of pulverized coal in power stations. The cementation effect of PFA relies on the formation of CSH which slowly hardens to form a stable material that may be similar to those of PC [45].

As discussed earlier, conventional barrier designs are primarily based upon achieving a low hydraulic conductivity to inhibit contaminated groundwater advective flow, without consideration of diffusive transport. However, low hydraulic conductivity, in itself, may not be sufficient to ensure that a barrier wall will effectively inhibit contaminant transport over a long period (e.g., decades) as the barriers typically have only a limited exchange/adsorption capacity for contaminants [46]. Some recent studies have incorporated reactive additives to deal with diffusive contaminant transport in low-hydraulic conductivity barriers while maintaining their desirable characteristic of low hydraulic conductivity. These barrier systems have been termed impermeable reactive barriers [47].

Because of their hydrophilic character, unmodified bentonites have only a limited ability to adsorb organic contaminants [48,49]. However, bentonites are receptive to be modified by exchanging inorganic cations with various types of organic cation [50]. Organoclay (OC) is a clay that has been modified to make it organophilic so that organic contaminants will sorb to it, and, therefore, be immobilized. Many organic cations may be used to modify bentonite, the most commonly used substances are quaternary ammonium cations (QACs). The QACs are surfactants consisting of an ammonium center with four branches for the attachment of functional groups [51]. Zeolites are naturally occurring or engineered aluminosilicates with an open, rigid, three-dimensional cage-like structure that contains channels and cavities. Contaminant metal ions that pass through the structure are trapped by ion-exchange reactions, in particular, zeolite is suited to adsorption of ammonia and heavy metal contaminants. Their cavity sizes enable the selective adsorption of some molecules into the porous structure while rejecting others on the basis of their size, giving zeolites their ‘molecular sieve’ title [52]. However, their efficiency toward organic contaminants is thought to be low due to their low organic carbon content [53]. Research has been conducted on the effect of zeolite in cement-based grouts for the deep mixing of clays, including on the mechanical performance, hydraulic conductivity, and durability for ground improvement purposes [21]. Zeolites have been blended with cement as they have been shown to offer strength as well as durability advantages over cement alone.

The in situ hydraulic conductivity is dependent on the homogeneity and quality of the mixed soil achieved by the contractor’s equipment and mixing methodology. The quality and homogeneity of soil mixing are governed by many factors, including the homogeneity, plasticity, and density of the in situ soil, as well as groundwater conditions, etc. [54]. The great variability of natural soils and the actual performance of the soil mixing equipment make it difficult to predict the final in situ hydraulic conductivity. The actual hydraulic conductivity may exhibit variation spatially, both horizontally and vertically, which tends to decrease the certainty in estimating the overall hydraulic conductivity of the cut-off wall. The UCS test is the most common test for assessing the strength of cement-mixed soil. The UCS of mixed soil depends on the type of the binder used, the geotechnical and chemical properties of the in situ soil [55]. Bruce and Bruce (2003) summarized the typical ranges of some key properties of cement-mixed soil [56]. The hydraulic conductivity ranges from 1 × 10^−6^ to 1 × 10^−9^ m/s, and the 28-day UCS from 0.5 to 5 MPa for granular soils and 0.2 to 2 MPa for cohesive soils.

## 3. Damage and Durability

**Figure 1:**
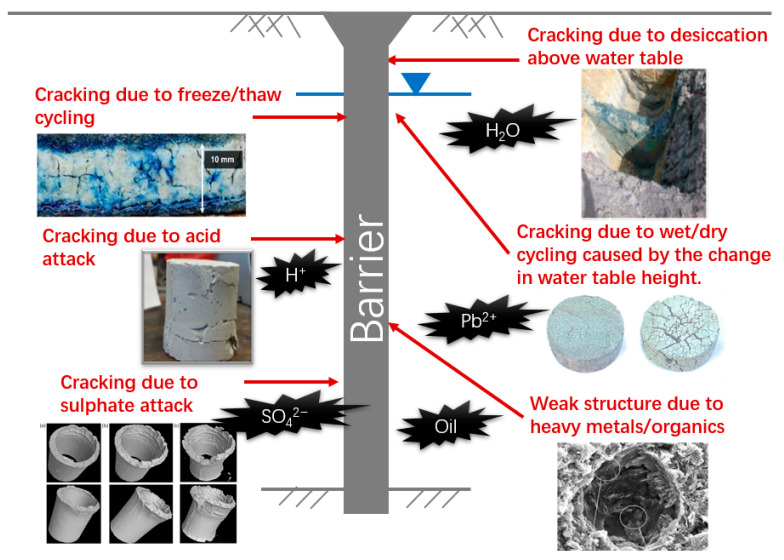
Cementitious barrier materials deteriorate under mechanical, chemical, and environmental stresses.

Despite the extensive use of vertical barriers in contaminated sites, the barrier materials deteriorate under mechanical, chemical, and environmental stresses (Figure 1). The damage can lead to problems related to the undermined mechanical and transport properties, impacting serviceability and reliability, and, in some cases, leading to an undetected physical breach of barriers.

### 3.1. Load-Induced Cracking

Although the primary function of barriers is to hinder the migration of contaminants and not to transmit load, it is often inevitable that they will experience changes in loading conditions throughout their service lives. For example, in areas that are susceptible to seismic activity, barriers could be attacked by earthquakes: micro and even visible cracks could take place, large deformations could develop, and, as a result, hydraulic conductivity may drastically increase [57]. The relationship between the deformation response and hydraulic conductivity of cement–bentonite in a triaxial cell was investigated [58]. The hydraulic conductivity immediately increased once the peak stress was exceeded (by 0.2% axial strain) to 1.3 × 10^−8^ m/s compared with the hydraulic conductivity of 4.6 × 10^−9^ m/s for the intact samples. The values were the highest at the post-peak state after exceeding 2.2% axial strain; they were of the 10^−6^ and 10^−7^ m/s order of magnitudes.

In addition to the laboratory studies, a forensic investigation reported a case study of a cement–bentonite barrier failure due to the change in load in Australia [59]. The designed function of the cement–bentonite wall was to limit water ingress to an excavation site. However, as the excavation progressed the barrier moved towards the excavation and the extent of this movement in the center of the wall was noted to be up to approximately 300 mm, which led to cracking and failure. Through the back analysis, it was understood that the increase in the depth of excavation and dewatering inside the chamber resulted in greatly increased deflections and the development of tensile stresses of the barrier.

### 3.2. Chemical Attacks in Contaminated Land

There is concern that groundwater contaminant plumes in some polluted sites may affect the performance of a barrier adversely. Sulfate attack in cement is characterized by expansion, causing loss of strength and stiffness, cracking, spalling, and eventual disintegration. Although no single reaction is responsible for all this expansion, it is generally agreed that the formation of gypsum (CaSO_4_·2H_2_O) and ettringite (calcium sulfoaluminate, 3CaO·Al_2_O_3_·3CaSO_4_·32H_2_O) is linked to expansion [60]. Joshi (2009) presented an immersion test of 11-year-old cement–bentonite barrier material in sulfate solution, and it was found that all samples had to some extent cracked, swelled, and become soft to the touch and lighter in color [39]. Additionally, acids dissolve both hydrated and unhydrated cement compounds, demolishing their crystalline structure and leaving incoherent residue [61]. Osman (2007) carried out an experimental study to investigate the impact of the acidic environment on cement mixed soils in terms of the change in the UCS and hydraulic conductivity, and it was concluded that soil stabilized with cement-based grouts are susceptible to the acid solution [62].

Because of the chemical bonding, hydrogen bonding, electrostatic force, and hydrophobic force, organic contaminants could have an affinity toward cement particles and cement hydration products [63]. Fernandez and Quigley (1985) presented the results of hydraulic conductivity tests on natural soil [64]. Clay samples mixed with pure liquid hydrocarbon showed a large range in hydraulic conductivity values from 10^−8^ m/s for water to 10^−4^ m/s for simple aromatics. Sequential permeation of water-saturated clay specimens with alcohol, and then simple aromatics (benzene, xylene, and cyclohexane) resulted in an increase in hydraulic conductivity by approximately three orders of magnitude. This sequential permeation leads to significant contraction of the diffuse electric double layer and pronounced increases in both micro- and macro-voids in the soil, leading to a drastic increase in hydraulic conductivity.

### 3.3. Damage Due to Aggressive Environments

In many regions in Asia, Europe, and North America, soil and geotechnical infrastructure are frequently subjected to freezing and frost heaving in the winter, and thaw and weakening during summer [65]. The hydraulic conductivity of the soil and cement-mixed soil can increase considerably when subjected to freeze–thaw cycles, usually with the first cycle causing the greatest damage [66,67]. Jamshidi et al. (2011) reported that an increase of up to two orders of magnitude in the hydraulic conductivity of cement-mixed soil can be observed after four freeze–thaw cycles [68].

When the soil and cementitious materials are exposed to desiccation, a zone of negative water pressure appears, and once tension forces exceed the tensile strength of the materials, shrinkage and cracking initiates [69]. Tedd (2005) found that hardened cement–bentonite material is susceptible to drying shrinkage, cracking, and even disintegration [70]. Ratnam (2002) also reported that cement–bentonite samples formed cracks within 24 h when allowed to air dry and disintegrated within 5 days. The drying process is irreversible, such that immersion of dried samples in water does not return them to their original state [71]. Cermak et al. (2012) explained that shrinkage has a great impact on stress states in barrier materials [72]. In the vertical and longitudinal directions, the continuous and homogeneous nature of the barrier and friction on the barrier sides offer restraint, and, therefore, the shrinkage can lead to a decrease in the compressive stress and possible development of tensile stress. In the longitudinal direction, where only barrier sides offer restraint, shrinkage can induce the most significant change in the stress state. It is found that at the bottom of the barrier, the longitudinal stress remains compressive; however, the calculated stress becomes tensile at the top part of the barrier, with the use of experimental values of the elastic modulus of the barrier material and a linear strain value of approximately 1.5 mm/m due to hydration shrinkage.

## 4. Case Studies

A number of landmark remediation projects demonstrate the effectiveness of the use of soil–bentonite, cement–bentonite, and soil mix barrier systems. Jefferis (1997) has given a comprehensive review of the engineering applications of slurry trench cut-off walls [22]. The field-scale applications of soil mix walls include the Ardeer site project in 1995 [73], West Drayton site project in 1997 [74], Long Eaton, Nottingham, project in 2000 [75], the Sir John Rogerson’s Quay, Dublin, project in 2004 [76], and the SMiRT project [21].

### 4.1. West Drayton Field Trials (1994–1995)

The site was described as an old chemical works and consisted of 1.7 m of made ground underlain by 3–4 m of natural sand and gravel on top of London clay. The groundwater table was at a depth of ~2 m. The soil and groundwater were contaminated by a mixture of heavy metals and organic contaminants, including concentrations of Pb and Cu of up to 3 g/kg, and total petroleum hydrocarbons (TPH) up to 9 g/kg. The mixes consisted of 75–84% soil, 1–7% PC, 0–16% PFA, 0–0.5% lime, 0–1% bentonite, and 3–13% water (percentages by mass), as well as a small amount of modified bentonite. A grid of 23 overlapping columns was formed over a period of two days, treating a plan area of 2.4 m × 2.4 m and a volume of 14 m^3^. Coring of the treated soil after two months showed that the treated soil was acceptably homogenous and consistent. The cores were tested against the criteria and were satisfactory [77]. The UCS ranged between 990 kPa and 1480 kPa, the hydraulic conductivity between 0.64 × 10^−9^ m/s and 2.56 × 10^−9^ m/s, and the leachate pH between pH 9.6 and pH 10.9. The project was successful in the development and implementation of the treatment. It highlighted the complex issues associated with full scale testing in terms of site heterogeneity. It revealed a complex time-dependent behavior of treated contaminated soil and found that it was difficult to isolate the effects of chemical and mechanical factors.

Further cores were extracted 4.5 years after treatment to examine the longer-term performance [75]. The 4.5-year-old cores showed little sign of deterioration compared to the earlier cores, with the UCS ranging between 1.4 MPa and 7.5 MPa, the set hydraulic conductivity between 0.01 × 10^−9^ m/s and 2.5 × 10^−9^ m/s, and the toxicity characteristic leaching procedure (TCLP) leachate between pH 6.3 and pH 7.4. A comparison between single and overlap columns showed that the overlap columns were not effectively mixed and contained lumps of single material surrounded by grout. The cores from the sand and gravel soil were more uniform than in the made ground soil due to extraneous materials.

### 4.2. West Drayton Commercial Project (1997)

Following the success of the West Drayton research project (1994–1995) above, commercial companies May Gurney and Envirotreat used soil mixing technology to remediate another site in West Drayton, a former paints factory contaminated with organics, heavy metals, and other inorganics. The grout mixture used on site was based upon a treatability study, the ratios used were cement: bentonite 1–2.5:1, soil: grout 3–6:1, and water: solids 3–6:1. A total of 4500 overlapping 600 mm and 900 mm diameter columns were installed over eight weeks. Throughout the construction, leaching tests were performed on the treated soil which showed the TPH concentration was below the criteria used. Subsequent groundwater monitoring over a two-year period also showed the treatment to be successful [42].

### 4.3. Sir John Rogerson’s Quay (2004)

The geology consisted of 1–4.5 m of made ground, mainly of sandy clay with gravel, cobbles, and ash. Medium-dense alluvial gravel containing perched water were present at a depth from 4.5–8.5 m. Elevated concentrations of aromatic hydrocarbons benzene and phenol and polyaromatic hydrocarbons naphthalene and benzo(a)pyrene were present in both lithologies, in both soil and ground water [76].

The remedial approach included the use of soil mixing technology to construct an impermeable barrier wall around the site, as well as solidification/stabilization (S/S) treatment. Because the treated soil had to satisfy a variety of environmental and geotechnical performance criteria, a pilot trial was undertaken using a number of different grout mixtures. Hydraulic conductivity, UCS, and leachate tests were carried out on various samples taken from the trial mixes. The results obtained from the trial enabled the final treatment grout mixtures to be confirmed. The two grout mixtures selected incorporated PC and both natural and modified bentonite. Testing was undertaken on 28-day-old samples on a regular basis; for the impermeable barrier, hydraulic conductivity tests were carried out for every 100 m^3^ of soil treated. Leaching and UCS tests were performed for every 300 m^3^ of soil treated. The hydraulic conductivity of the barriers was between 0.11 and 0.94 × 10^−9^ m/s and the UCS was from 0.73 to 4.14 MPa. Although there was variability in the results, the treatment satisfied the target criteria. The variability was generally thought to be due to homogeneity, both in the variety and concentrations of contaminants in the site soil.

### 4.4. SMiRT Project

Project SMiRT (Soil Mix Remediation Technology) was conducted in May 2011, at a contaminated site in Castleford, West Yorkshire, UK. The project tasks involved laboratory treatability studies and field trials (field testing and monitoring and laboratory testing of site cores) [21]. The construction of the impermeable barrier system was such that six barrier wall sections were installed to form a hexagon, each wall was ~8 m long denoted I1 to I6. The triple columns were overlapped by 0.1 m within the augers and then each installation was overlapped by 0.2 m. Homogeneity was examined for the six SMiRT barrier walls by assessing the quality of retrieved site cores. Above a 3 m depth, the mean total core recovery (TCR = length of core/total length of core run) for all barrier walls was 92%, and 52% of the core runs were recovered as solid intact cores, and the mean rock quality designation (RDQ = length of core sticks ≥100 mm/total length of core run) was 75%. Hence, in general, the cores obtained from the top 3 m demonstrated good core recovery parameter values, indicating a good degree of mixing between the grout and the soil to this depth. At a depth greater than 3 m, the mean TCR for all the barrier walls fell to 75% and only 23% of the core runs were recovered as solid intact cylindrical cores (less than half the value in the top 3 m), the mean RQD fell to 48%. The reason for this is thought to be caused by a number of influencing factors, including: (i) the difficulty in the deep mixing of clays/silts present in the layer between the made ground and sand and gravel, which sometimes form untreated lumps, (ii) the groundwater diluting the grout, leading to low strength, (iii) the mixing of the three soils zones (made ground/silt layer/sand and gravel) leading to the increased heterogeneity in the mixing process, (iv) contamination in the groundwater affecting the cement hydration process, and (v) reduced binder content due to poor on-site construction/quality control.

The core analysis found that there are clear trends with depths between wall sections and with generally good performance at 3 years. Core tests found strength values of approximately 2.5 MPa. The mean UCS of the barrier walls ranged from 0.67 MPa (I5) to 3.96 MPa (I3) and a distinctly lower mean strength was observed for walls I1 and I5. In general, the SMiRT barrier wall results showed a fairly low amount of variation, again showing a good degree of mixing in comparison to other projects. However, there was an apparent lack of cement grout mixed into the soil within the I1 and I5 barriers, attributed to defects due to issues with on-site construction. The mean hydraulic conductivity of the barrier wall cores ranged from 8.55 × 10^−9^ m/s (I3) to 4.65 × 10^−7^ m/s (I5). A distinctly greater hydraulic conductivity was observed for core specimens from walls I1 and I5, reflecting the poor quality of the cores. Based on a cement content used in the SMiRT barrier walls, it would be expected that SMT barrier walls would have hydraulic conductivity of 1 × 10^−8^ m/s.

Overall, the site cores containing PC and GGBS performed better than the cores containing CEM IV in terms of hydraulic conductivity, the cores containing zeolite performed better than those containing bentonite, and no effect was seen with the addition of organoclay, though this assessment was made more difficult by the poor performance of walls I1 and I5. Overall, it was shown that the I3 grout mixture performed best throughout the tests; it is the combination of PC-GGBS with zeolite that brought about the lowest hydraulic conductivity, highest UCS, and lowest leachability. Therefore, it is concluded that the use of PC-GGBS–Zeolite grout mixture is recommended for future barrier wall construction.

## 5. Resilient Materials and Performance Monitoring

### 5.1. Crack-Resistant and Self-Healing Materials

Traditionally, infrastructure materials have been designed to meet fixed specifications, and material deterioration has been regarded as inevitable. However, recently, inspired by biological systems, the development of smart and resilient materials has gained increasing attention. The majority of the studies and applications to date have been focused on self-healing cement paste, mortar, and concrete for structural applications, with significant advances being achieved [78,79,80]. Despite the advances in self-healing structural materials, to the best of the authors’ knowledge, very little attention has been given to the incorporation of such concepts in in-ground barrier applications. The EPSRC-funded Resilient Materials for Life (RM4L) project has been carried out in collaboration between the Universities of Cambridge, Cardiff, Bath, and Bradford along with more than 20 international industrial partners to develop smart infrastructure materials. The concept of introducing crack-resistant and self-healing systems in impermeable barrier materials has emerged as part of this project.

**Figure 2:**
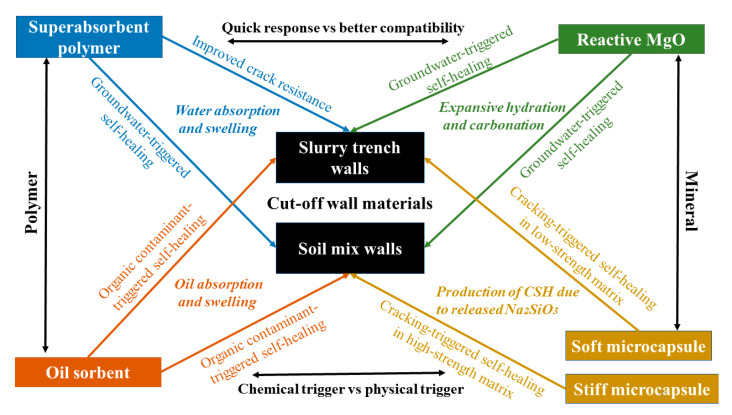
Schematic of work carried out at Cambridge on different polymer and mineral additives in slurry trench and soil mix barrier materials.

The Geotechnical and Environmental Research Group at Cambridge has developed crack-resistant and self-healing impermeable barrier materials incorporating polymers and minerals. The overall performance of four additives, including superabsorbent polymers (SAPs), oil sorbent polymers, reactive MgO pellets, and microencapsulated sodium silicate in two impermeable barrier materials (cement–bentonite slurry and cement mixed soil) is the interest of the research [81,82,83,84]. The schematic of work carried out on different polymer and mineral additives is shown in Figure 2. Each healing agent has its own advantages and disadvantages in different application scenarios, and, therefore, a comparison between these systems is made.

SAPs are only responsive to water solutions and the absorption and swelling can be triggered quickly by the ingress of groundwater in polluted land. In organically polluted sites, oil sorbents can be triggered by organic contaminants and block the crack to prevent further chemical attack. The self-healing performance of these two polymers in barrier materials is significant in terms of the recovery of hydraulic conductivity. In soil mix samples, the post-healing hydraulic conductivity of SAP-containing samples was only slightly higher than the undamaged values. For oil sorbent-containing cement–bentonite and soil mix samples, the post-healing permeabilities were both reduced by nearly an order of magnitude compared with the control samples. This means SAPs and oil sorbents can swell and block the cracks when triggered by the ingress of contaminant liquids. However, the effects of SAPs and oil sorbents on the UCS are different. SAPs absorb mixing water, and, therefore, reduce the water-to-cement ratio of cement–bentonite, and, as a result, the UCS increased with the increasing SAP dosage by up to 23% compared with the control samples. In contrast, the addition of oil sorbents decreased the UCS of cement–bentonite samples by 9% and of soil mix ones by 23%. Despite the adverse effect of oil sorbents, the UCS of the barrier materials were still higher than the required values. Furthermore, both SAPs and oil sorbents increased the strain at failure of the barrier materials significantly. The addition of these tensible polymers improves the ductility of the cementitious matrix, which reduces the possibility of brittle fracture and is beneficial to barriers.

Furthermore, the mineral additives such as MgO pellets and microencapsulated sodium silicate were investigated to complement the abovementioned polymer healing agents. Although SAPs and oil sorbents can swell and block cracks very effectively and quickly, these swollen polymers are not compatible with the cementitious materials and are unable to provide strength. The mineral healing products of MgO and sodium silicate microcapsules, however, are more compatible with the cementitious matrix, and, thus, can potentially provide strength recovery. The expansive hydration and carbonation reactions of reactive MgO are triggered by the ingress of groundwater and dissolved CO_2_. The addition of MgO pellets decreased the post-healing hydraulic conductivity values by almost an order of magnitude in cement–bentonite samples and by approximately half of an order of magnitude in soil mix samples compared with the control ones. The effects of MgO pellets, regardless of the size, on the engineering properties of barrier materials are slight or negligible. Neither brucite nor hydrated magnesium carbonates are a part of the hydration products of cement. Sodium silicate, therefore, is considered a more compatible mineral candidate for the self-healing of cement-based barrier materials. Sodium silicate reacts with calcium hydroxide in the presence of water to form a CSH gel—the main product of cement hydration. The post-healing hydraulic conductivity decreased by more than half of an order of magnitude when 4% soft microcapsules were added in low-strength cement–bentonite samples compared to the control ones. Additionally, the recovered hydraulic conductivity of stiff microcapsule-containing soil mix samples was only slightly higher than the undamaged values. In general, the addition of microcapsules has a slightly adverse or negligible effect on the mechanical properties of the cement–bentonite and soil mix samples, making them a suitable healing agent for barrier materials.

The successful development of such crack-resistant and self-healing impermeable barrier materials has the potential to yield substantial repair and maintenance savings and to enhance the durability and serviceability of geo-environmental applications.

### 5.2. Performance Monitoring Techniques

One of the most important considerations for the assessment of in-ground barriers is to demonstrate that the barrier wall is homogeneous and free of defects. However, this cannot be answered conclusively based on the current state of practice. Heterogeneities within the treated soil inevitably exist at the time of construction due to factors such as incomplete mixing and the presence of any extraneous materials that may be present in the soil. This variability is not easily investigated, and the in situ hydraulic conductivity of barrier walls is often assumed to equate to laboratory-tested specimens created from remolded bulk samples. However, remolded bulk samples are obtained in the field before the wall sets and may not reflect the true nature of the barrier wall from which the sample came. In one case study [26], hydraulic conductivity testing was performed on samples recovered from within a soil–bentonite slurry trench wall, considered suspect due to a high sand content near the base of the wall. The study found that, although the remolded specimens met the project criteria in the laboratory, the in situ material near the base of the wall was substantially coarser, and only a few defects could significantly increase the bulk hydraulic conductivity. Moreover, laboratory hydraulic conductivity values may not be representative of the in situ hydraulic conductivity if the applied stress state in the test is not representative of the in situ stress state. These data, while limited, point to the need for more comprehensive in situ investigations of in-ground barriers.

Post-construction monitoring of barrier walls is rarely performed and typically involves monitoring of the aquifer downgradient of the barrier rather than an actual assessment of the wall itself by coring or in situ testing. Coring and in situ hydraulic conductivity test methods include (1) laboratory testing of core samples from the constructed barrier, (2) in situ falling and/or rising head tests (i.e., slug tests) performed in a borehole within the barrier, (3) in situ piezocone soundings with pore pressure dissipation measurements, and (4) pump tests using wells installed adjacent to the barrier [85,86]. In situ methods, although rarely used, offer the great advantage of testing a larger and more representative volume of the barrier materials. Small and convenient devices such as the piezocone could be used in the field; however, larger-scale tests can be more representative [34]. For example, slug tests can be conducted by constructing a vertical well in the barrier wall [86]. The field piezocone test used to evaluate the hydraulic conductivity of cementitious vertical barriers would usually yield cracks and therefore may overestimate the hydraulic conductivity. An instantaneous change in the hydraulic head of the well is made by quickly adding or removing a known volume of water. The rate of the hydraulic head increases or decreases in the well is monitored until the water level returns to the static condition, and hydraulic conductivity is calculated from the test results.

Core samples are typically collected by drilling into the barrier to the desired depth. Laboratory core specimens are relatively small (60 to 100 mm diameter) and are less likely to capture defects and other macro features that often govern hydraulic conductivity at the field scale [87]. Therefore, laboratory tests may yield hydraulic conductivity values lower than those from in situ tests that capture a larger representative volume of the barrier and are more likely to be influenced by macro features. Britton et al. (2004) compared the hydraulic conductivity of a soil–bentonite backfill in three pilot-scale barriers based on small-scale laboratory tests, piezometer tests, piezocone soundings, and large-scale pumping tests [85]. The results indicate that both remolding and the scale of the test sample size have a significant impact on the measured hydraulic conductivity in all of the three pilot-scale walls. In each case, small-scale laboratory tests on remolded samples returned the lowest hydraulic conductivity values, whereas the in situ tests consistently returned higher hydraulic conductivity values. These results highlight the importance of field testing to assess the hydraulic conductivity of a constructed barrier wall. 

The homogeneity of soil–bentonite and cement-based materials is crucial to the containment performance of barriers. For example, soil–bentonite windows would increase the breakthrough of target contaminants. The contact condition between the interface of keying and the aquitard primarily depends on the hydraulic conductivity and sorption capacity of the aquitard soil. Homogeneity can initially be evaluated by a visual observation of the cores and the determined rock quality designation value. Further evaluation can be conducted by assessing the UCS of cement-based samples and their corresponding coefficient of variation. If all the core recovery is used, then the average may be low, and the variance high due to disturbances. If the better cores specimens are selected, the average will probably be quite high, so the correct distribution is uncertain and should be assumed. However, the variability of UCS is not applicable for soil–bentonite homogeneity evaluation. The field electrical resistivity method may be suitable to capture local defects (e.g., windows) of soil–bentonite walls [88]. Researchers have found it difficult to estimate the influence of the uncertainties related to the strength of treated soil when testing core samples. As an example, Rogbeck (1997) presented UCS results from core samples, extracted columns, and penetration tests [89]. The UCS of core samples were significantly lower than that of whole column sections tested and the column penetration tests. When the strength of treated soil is relatively low, it has been found that only the best parts should be tested due to poor core recovery [87].

A few test methods can measure strength and deformation properties directly in situ; however, there is currently not enough experience from the pressure meter test or geophysical tests (such as electrical resistivity) for these methods to be seen as reliable at present, and additional research and development is needed. In situ penetration tests can also be used to test strength, where a probe penetrates the treated soil using a dropped hammer weight. The number of blows to reach a certain depth provides a rough index of the strength of the treated soil. The standard penetration test (SPT), and other equivalent tests such as the Dynamic Probe Super Heavy (DPSH) penetration test, may be the most widely used field test method for geotechnical site investigation and is regularly used to test soil mixing-treated soil. However, Larsson (2005) claimed that penetration test results must be regarded as coarse and used as a relative measure, and that these tests should only be used as a supplementary strength test method [90]. Therefore, it can be concluded that laboratory strength tests on core samples should be the primary test methods for the quality assessment of in-ground barriers for strength.

## 6. Future Perspectives

A wide range of future work needs to be conducted in order to advance the understanding and application of in-ground barriers and increase the confidence of geo-environmental engineers to adopt these approaches. Proposed future work needs to focus on two main areas: the use of geophysical methods for non-destructive monitoring and the optimization of resilient in-ground barrier materials.

Although in situ geophysical methods (e.g., electrical resistivity, electromagnetic, acoustic) provide the promise of cost-effective and non-destructive post-construction evaluation of flaws in in-ground barriers, only very limited successful case studies are available in the literature [91]. Improvements in several areas may help facilitate wider and more successful applications of geophysical methods to the monitoring of in-ground barrier systems, such as the (1) incorporation of geophysical methods into the design of monitoring plans at an early stage; (2) development of more convenient and advanced methods for data acquisition, mining, processing, and interpretation; (3) introduction of artificial intelligence algorithms into geophysics data analysis and development of data-driven solutions to improve the reliability; (4) development of novel instrumentation. For example, the interpretation of geophysical data of in-ground barriers may be more reliable and efficient if a background dataset of the in situ soil is available before the barrier construction [92]. Any changes in subsurface geophysical properties caused by contaminant transport or breakthrough could be detected through a comparison of monitoring data and background data.

The self-healing performance of SAPs, oil sorbents, MgO, and microcapsules has been investigated separately, and no combined systems of any these healing agents have been studied. Each healing agent has its own advantages and disadvantages. For example, SAPs are only responsive to water solutions, whereas oil sorbents are triggered by organic liquids. In a polluted site with both contaminated groundwater and organic contaminants, the self-healing performance of a barrier would be more effective with the combined system of SAPs and oil sorbents. Additionally, the mineral additives such as MgO pellets and microencapsulated sodium silicate can be used to complement the polymeric healing agents. SAPs and oil sorbents can swell and block cracks very quickly (usually within a few hours) when triggered by the ingress of contaminants; however, these swollen polymers are not compatible with the barrier materials and are unable to provide strength. In contrast, it takes weeks or even months for the mineral healing agents to yield enough healing products to heal cracks. These mineral healing products are compatible with the cementitious matrix, and, thus, can potentially provide strength recovery. Other novel materials such as engineered cementitious composites (ECC) and modified ECC have also been found to possess excellent self-healing performance of tensile-induced cracks upon hydration. The hydraulic conductivity values in water and target contaminant solutions of ECC and modified ECC have shown the good chemical compatibility of these materials [93,94]. The combination of these polymeric and mineral healing agents is expected to block cracks within several minutes and help the cementitious matrix regain some strength after several weeks or months.

The lack of standardized test methods for self-healing geo-environmental materials may hinder international collaboration and slow further development. Additionally, it can impede future commercialization as it is difficult to convince engineers, who are used to a strictly regulated construction methodology. Recently, six different inter-laboratory testing programs to evaluate test methods to assess the efficiency of self-healing concrete have been established within the framework of the EU COST Action SARCOS [95]. However, many of those established standardized test methods for concrete cannot be applied to barrier materials. Specialized standard geo-environmental laboratory test methods need to be established for advanced barrier materials. For example, a long-term triaxial cell hydraulic conductivity test can be used to monitor the recovery of the hydraulic conductivity of barrier materials in the long term, and a triaxial shear test can be used to measure the mechanical properties considering the effects of earth pressure, consolidation, and drained/undrained conditions. Finally, the laboratory-scale model barriers and large-scale field trials are needed to establish the efficacy of different additives and verify the proposed crack-resistant and self-healing approaches.

## Figures and Tables

**Table 1:** Summary of reported values of the cement, bentonite, water, and GGBS proportions in cement–bentonite barriers.

Reference	Cement (%)	Bentonite (%)	GGBS (%)	Water (%)	Water-to-Binder Ratio
Evans (1993) [17]	15–30	4–7	-	65–80	2.2–5.4
Manassero et al. (1995) [34]	7.7	4	11.5	76.8	4.0
Philips (2001) [35]	3.5	3.5	13	80	4.8
Opdyke and Evans (2005) [30]	1–20	4–4.5	0–18	76–85.5	3.8
Joshi et al. (2010) [18]	2.5	3.4	10.1	84	6.6
Carreto et al. (2016) [36]	12.6–16.2	2.8–2.9	-	81.0–84.3	5.0–6.6
Royal et al. (2017) [37]	3.2	3.2	12.9	80.7	5.0

## Data Availability

All data generated or used during the study appear in the submitted article.
